# A Substructure-Informed Surrogate Framework for Uncertainty-Based Low-Cycle Fatigue Reliability Assessment of Integrated Blisk–Shaft Rotors

**DOI:** 10.3390/ma19204404

**Published:** 2026-10-20

**Authors:** Hongyun Sun, Xinqi Li, Xinjie Bai, Wenruo Li, Huiqun Yuan, Hongyuan Zhang

**Affiliations:** 1 School of Automotive and Transportation, Shenyang Ligong University, Shenyang 110159, China; 15715380361@163.com (X.L.); 17832875307@163.com (X.B.);; 2 College of Sciences, Northeastern University, Shenyang 110819, China; yuan_hq@163.com

**Keywords:** integrated blisk–shaft rotor, low-cycle fatigue, artificial neural network, uncertainty quantification, surrogate modeling, fatigue reliability

## Abstract

Integrated blisk–shaft rotors are susceptible to low-cycle fatigue (LCF) under complex operating conditions and parameter uncertainties, rendering direct finite-element-based probabilistic assessment computationally demanding. This study develops a surrogate-assisted fatigue-reliability framework integrating dominant-cycle identification, an improved cat swarm optimization-assisted artificial neural network (ICSO-ANN), and Monte Carlo simulation. Elastoplastic finite-element analysis is first performed to identify the fatigue-critical region and dominant fatigue cycle. Subsequently, 1000 finite-element samples are generated using Latin hypercube sampling. Four independent assessments are conducted, each using 200 samples for model development and 50 held-out samples for independent testing. The ICSO-ANN achieves a MAPE of 0.77%, demonstrating high accuracy in fatigue life prediction. Probabilistic analysis against a normally distributed design life requirement with a mean of 11,000 cycles yields a reliability of approximately 98.6%. On an identical 10^3^-sample basis, the surrogate-assisted analysis achieves an approximately 69-fold computational speedup over direct finite-element-based Monte Carlo analysis. Sensitivity analysis further identifies fatigue strength parameters and rotational speed as the dominant contributors to fatigue life uncertainty. The proposed framework provides a computationally efficient approach for the uncertainty propagation and probabilistic LCF reliability assessment of integrated blisk–shaft rotors.

## 1. Introduction

Integrated blisk–shaft rotors have gained increasing attention in advanced aeroengines owing to their advantages in weight reduction, structural integration, and improved dynamic performance [1,2,3]. By eliminating conventional mechanical joints, such as firtree attachments and bolts, the integrated configuration enhances load transmission efficiency and reduces structural mass [4,5,6]. However, the high degree of structural integration also intensifies the coupling among the blades, disks, and shaft, resulting in more complex dynamic interactions and pronounced local stress concentrations. Under high rotational speeds and varying aerodynamic loads, critical regions of the integrated rotor are prone to severe stress gradients and localized deformation, which may significantly affect structural integrity and service reliability [7,8,9,10].

Low-cycle fatigue (LCF) is one of the dominant failure mechanisms of aeroengine rotor components subjected to repeated start-up, acceleration, cruise, and shutdown processes [11,12,13]. For integrated blisk–shaft rotors, strong structural coupling may intensify local stress–strain concentrations and promote elastoplastic deformation under multiple operating conditions. Consequently, fatigue damage accumulates over representative loading cycles, with the relative contribution of each cycle governing the overall fatigue response. Deterministic elastoplastic finite-element analysis, coupled with strain–life relationships incorporating mean stress correction and cumulative damage criteria, therefore provides an effective basis for fatigue life prediction, critical-region identification, and dominant-cycle assessment [14,15,16]. More broadly, recent studies have demonstrated the effectiveness of numerical modeling, including finite and discrete element approaches, in characterizing localized mechanical responses and failure behavior, with experimental–numerical comparisons providing an important basis for assessing model credibility [17,18,19]. Despite their effectiveness in characterizing local elastoplastic responses and fatigue damage, deterministic approaches cannot adequately account for the inherent variability arising from uncertainties in material properties, loading conditions, geometric parameters, and fatigue-related material parameters. Consequently, a single deterministic fatigue life estimate cannot fully characterize the variability and reliability of the fatigue performance of integrated rotor systems [20,21].

To account for uncertainty-induced dispersion in fatigue life, probabilistic fatigue analysis and reliability assessment have been extensively performed for turbine blades, disks, and blisk systems [22,23,24,25]. Refs. [26,27] primarily incorporated stochastic material and loading parameters into strain–life models to evaluate the statistical distribution of fatigue life. To alleviate the computational burden associated with complex structures, surrogate modeling techniques, including response surface methods, neural networks, and ensemble learning, have been increasingly employed to approximate the nonlinear mapping between uncertain inputs and fatigue responses [24,28,29]. Substructure-based strategies have also demonstrated considerable potential for use in the efficient probabilistic analysis of multi-component systems. For example, Gao et al. proposed a distributed collaborative probabilistic framework for blade–disk systems, combining substructure simulation and response surface modeling to achieve efficient reliability evaluation [23]. More recently, deep learning-based surrogate models have been applied to fatigue reliability problems, demonstrating improved predictive capability in high-dimensional and strongly nonlinear scenarios [30,31]. Nevertheless, existing studies have predominantly focused on individual components or blade–disk assemblies, whereas systematic investigations into the fatigue reliability of multi-stage integrated blisk–shaft rotor systems remain limited.

Beyond the structural complexity of integrated rotor systems, a further challenge arises from the treatment of multiple operating cycles in probabilistic fatigue assessment. Direct Monte Carlo simulation (MCM) coupled with nonlinear elastoplastic finite element analysis becomes computationally demanding when uncertainties are propagated across multiple load cycles [29,32,33,34]. Although fatigue damage may be accumulated from several representative operating conditions, not all load cycles contribute equally to the total damage. Existing approaches often attempt to evaluate fatigue reliability across all cycles, leading to excessive computational burden without necessarily improving physical insight. Therefore, an efficient strategy is required to bridge deterministic multi-cycle fatigue analysis and probabilistic reliability assessment by focusing on the dominant fatigue mechanism.

Motivated by these considerations, this study proposes a critical-cycle-oriented fatigue reliability framework for an integrated blisk–shaft rotor. First, a substructure-informed finite element model is established to characterize the local elastoplastic responses and identify fatigue-critical regions. Deterministic low-cycle fatigue analyses are then performed under three representative load cycles, and the dominant fatigue cycle is identified according to its damage contribution. Subsequently, an improved cat swarm optimization-based artificial neural network (ICSO-ANN) surrogate model is developed to establish the nonlinear mapping between uncertain parameters and fatigue life under the dominant cycle. The validated surrogate model is further integrated with Monte Carlo simulation to efficiently quantify fatigue life uncertainty, failure probability, and structural reliability, while avoiding repeated nonlinear finite element analyses. Finally, sensitivity analysis is conducted to quantify the relative influence of uncertain parameters on fatigue life and identify the dominant sources of fatigue life variability.

The remainder of this paper is organized as follows. Section 2 presents the proposed methodology and theoretical formulation. Section 3 describes the integrated rotor model and loading conditions, and provides deterministic fatigue analysis and dominant cycle identification. Section 4 presents surrogate modeling, reliability evaluation, and sensitivity analysis. Conclusions are drawn in Section 5.

## 2. Critical-Cycle-Oriented Fatigue Reliability Methodology

### 2.1. Overall Framework of the Proposed Method

**Figure 1:**
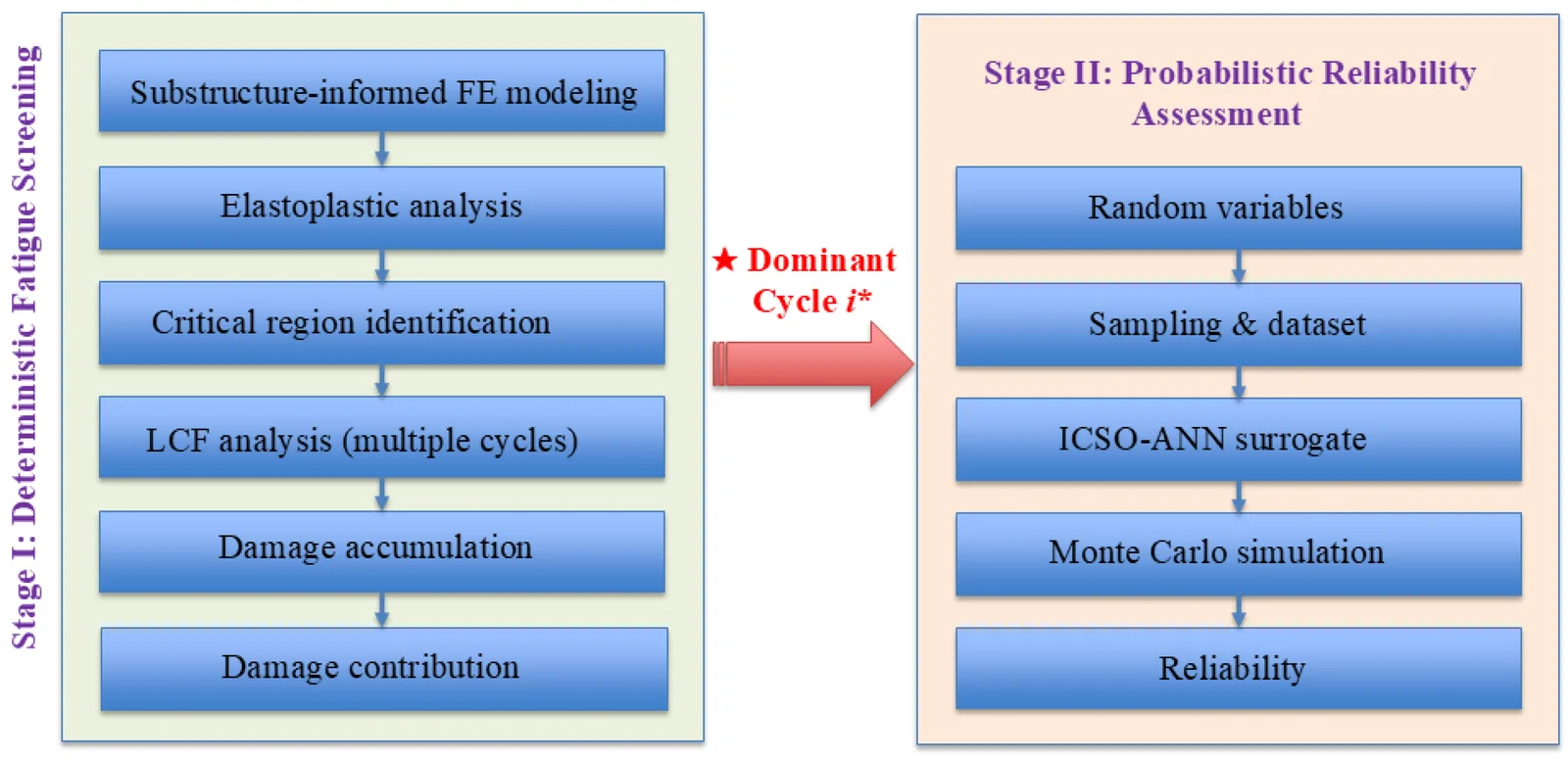
Overall framework of the proposed critical-cycle-oriented fatigue reliability assessment method.

This study proposes a critical-cycle-oriented fatigue reliability framework for an integrated Blisk–Shaft rotor. Since different operating cycles contribute unequally to fatigue damage, a two-stage strategy is adopted to balance computational efficiency and physical fidelity. The overall procedure is illustrated in Figure 1.

In the first stage, a substructure-informed finite element model is established for the integrated rotor system. Elastoplastic analyses are performed under representative operating conditions to obtain the local stress–strain responses in critical regions. Deterministic low-cycle fatigue analyses are then conducted under three representative load cycles. Based on the corresponding fatigue damage results, the dominant fatigue cycle is identified according to the damage contribution ratio. In this way, the multi-cycle fatigue problem is transformed into a dominant-cycle-oriented reliability problem.

In the second stage, probabilistic fatigue reliability assessment is performed for the dominant fatigue cycle. Material parameters, loading parameters, and fatigue properties are treated as random variables. An ICSO-optimized artificial neural network surrogate model is constructed to approximate the nonlinear relationship between uncertain inputs and fatigue life. The trained surrogate model is subsequently combined with Monte Carlo simulation to evaluate fatigue life distribution, failure probability, and reliability efficiently.

### 2.2. Substructure-Informed Modeling

To efficiently analyze the integrated blisk–shaft rotor, a substructure-informed finite element modeling strategy is adopted. The rotor is first regarded as a coupled structural system consisting of multiple blade–disk stages and the shaft. According to the geometric and mechanical characteristics of the rotor, the global structure is decomposed into several stage-wise blade–disk substructures and shaft-related substructures. This treatment facilitates mesh generation, interface coupling, and local response extraction while preserving the mechanical interaction among different rotor components.

For the integrated rotor system, the static equilibrium equation can be expressed as

(1) Ku=F

where K is the global stiffness matrix, u is the nodal displacement vector, and F is the external load vector. In the substructure formulation, the degrees of freedom of each substructure can be divided into retained interface degrees of freedom and internal degrees of freedom. The equilibrium equation of a substructure can be written as

(2) $$\left[\begin{array}{ll} K_{bb} & K_{bi}\\ K_{ib} & K_{ii} \end{array}\right]\left[\begin{array}{l} u_{b}\\ u_{i} \end{array}\right]=\left[\begin{array}{l} F_{b}\\ F_{i} \end{array}\right]$$

where the subscripts *b* and *i* denote the interface and internal degrees of freedom, respectively. By condensing the internal degrees of freedom, the reduced equilibrium equation of the substructure can be obtained as

(3) $$K^{*}u_{b}=F^{*}$$

with

(4) $$\begin{array}{l} K^{*}=K_{bb}-K_{bi}K_{ii}^{-1}K_{ib},\\ F^{*}=F_{b}-K_{bi}K_{ii}^{-1}F_{i}. \end{array}$$

Here, $K^{*}$ and $F^{*}$ are the condensed stiffness matrix and equivalent load vector of the substructure, respectively. This formulation provides the theoretical basis for reducing the computational scale of large structural systems while maintaining the coupling relationship at substructure interfaces.

In the present work, the substructure concept is mainly used to guide the stage-wise finite element modeling and local response extraction of the integrated blisk–shaft rotor. Each blade–disk stage is treated as a structural subdomain, and adjacent substructures are connected through compatible interface nodes to ensure displacement continuity and load transfer. Since different blade–disk stages may have different blade numbers and geometric features, the substructure-informed strategy improves the flexibility of mesh generation and avoids excessive mesh distortion in the global model.

### 2.3. Elastoplastic and Low-Cycle Fatigue Model

The integrated blisk–shaft rotor may experience local elastoplastic deformation under high-speed operating conditions. Therefore, elastoplastic stress–strain analysis is required before fatigue life evaluation. In the present study, the equivalent stress is evaluated using the von Mises criterion,

(5) $$\sigma_{\mathrm{eq}}=\sqrt{\frac{1}{2}\left[{\left(\sigma_{1}-\sigma_{2}\right)}^{2}+{\left(\sigma_{2}-\sigma_{3}\right)}^{2}+{\left(\sigma_{1}-\sigma_{3}\right)}^{2}\right]},$$

where σ_1_, σ_2_, and σ_3_ are the three principal stresses. Local yielding is considered to occur when

(6) $$\sigma_{\mathrm{eq}}\geq \sigma_{y},$$

where σ_y_ is the yield strength of the material.

To describe the elastoplastic behavior of the rotor material, a bilinear isotropic hardening model is adopted. The stress–strain relationship can be written as

(7) $$\sigma =\left\{\begin{array}{ll} E\varepsilon , & \sigma <\sigma_{y}\\ \sigma_{y}+E_{t}\left(\varepsilon -\varepsilon_{y}\right), & \sigma \geq \sigma_{y} \end{array}\right.$$

where *E* is the elastic modulus, *E_t_* is the tangent modulus, *ε* is the strain, and ε_y_ is the yield strain. The bilinear hardening model accounts for the change in material stiffness after yielding, thereby enabling the local plastic deformation and associated stress redistribution to be captured in the fatigue-critical regions.

The low-cycle fatigue life is evaluated using the strain–life approach. Considering the influence of mean stress, the Morrow mean-stress-corrected Coffin–Manson model is employed,

(8) $$\frac{\Delta \varepsilon }{2}=\frac{\sigma_{f}^{\prime }-\sigma_{m}}{E}{\left(2N_{f}\right)}^{b}+\varepsilon_{f}^{\prime }{\left(2N_{f}\right)}^{c}$$

where Δε/2 is the strain amplitude, σ*_m_* is the mean stress, *N_f_* is the number of cycles to failure, $\sigma_{f}^{\prime }$ is the fatigue strength coefficient, *b* is the fatigue strength exponent, $\varepsilon_{f}^{\prime }$ is the fatigue ductility coefficient, and *c* is the fatigue ductility exponent. The strain amplitude and mean stress extracted from the elastoplastic finite element results are substituted into this model to calculate the fatigue life under each representative load cycle.

The fatigue damage induced by cyclic loading is evaluated using Miner’s linear cumulative damage rule. For the *i*-th representative load cycle, the corresponding fatigue damage is defined as

(9) $$D_{i}=\frac{n_{i}}{N_{i}}$$

where *n*_i_ and *N*_i_ denote the applied cycle number and the predicted fatigue life, respectively.

### 2.4. Critical-Cycle Identification

The integrated blisk–shaft rotor is subjected to different cyclic loading conditions during service. These cycles may generate different local stress–strain responses and, consequently, different fatigue damage levels. Since the subsequent probabilistic reliability analysis requires repeated fatigue life evaluations under random inputs, directly considering all representative cycles would result in a high computational burden. Therefore, a dominant-cycle identification criterion is introduced to select the most fatigue-critical cycle for reliability analysis.

Based on the deterministic fatigue results obtained from Section 2.3, the total fatigue damage caused by the three representative load cycles is expressed as

(10) $$D_{\mathrm{total}\;}=\sum_{i=1}^{k}D_{i}=\sum_{i=1}^{k}\frac{n_{i}}{N_{i}}$$

The damage contribution ratio of the *i*-th load cycle is defined as

(11) $$\eta_{i}=\frac{D_{i}}{D_{\mathrm{total}\;}}\times 100\%$$

where *η*_i_ represents the relative contribution of the *i*-th cycle to the total fatigue damage. A larger value of *η*_i_ indicates that the corresponding cycle plays a more dominant role in fatigue damage accumulation.

The dominant fatigue cycle is selected as the cycle with the maximum damage contribution,

(12) $$i^{*}=\arg\;\underset{i}{\max}\left(\eta_{i}\right)=\arg\;\underset{i}{\max}\left(D_{i}\right),$$

where *i*^∗^ denotes the index of the dominant fatigue cycle. The fatigue life and damage response associated with this cycle are then used as the target response for surrogate modeling and reliability assessment.

### 2.5. ANN Surrogate Model

For the dominant fatigue cycle identified in Section 2.4, direct elastoplastic finite element and fatigue life calculations under a large number of random samples would be computationally expensive. Therefore, an artificial neural network (ANN) surrogate model is introduced to approximate the nonlinear relationship between uncertain parameters and fatigue life.

**Figure 2:**
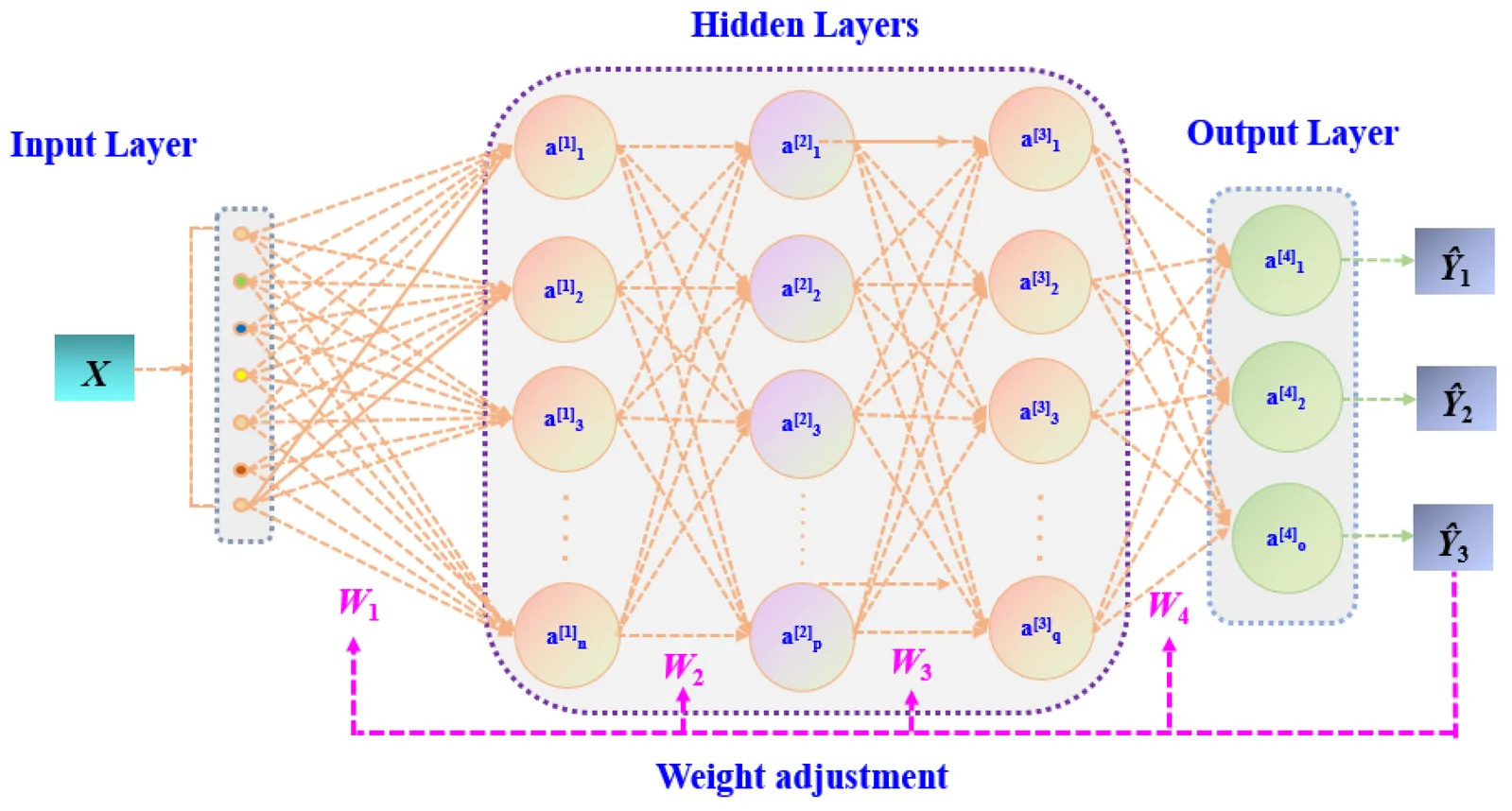
Architecture of the artificial neural network (ANN) surrogate model.

The input vector of the surrogate model is defined as

(13) $$X=[X_{1},X_{2},\ldots ,X_{m}],$$

where *X_j_* denotes the *j*-th random variable, including material parameters, loading parameters, and fatigue properties. The output is the fatigue life under the dominant fatigue cycle,

(14) $${\hat{N}}_{f}^{i^{*}}=f_{\mathrm{ANN}}(X;\theta ),$$

where ${\hat{N}}_{f}^{i^{*}}$ is the predicted fatigue life, $f_{\mathrm{ANN}}$ represents the nonlinear ANN mapping, and **θ** denotes the model parameters and hyperparameters. The schematic architecture of the ANN surrogate is shown in Figure 2.

The ANN is trained using input–output samples generated from deterministic finite element fatigue analyses. For each input sample *X_k_*, the local stress–strain response at the identified critical region is extracted, and the corresponding reference fatigue life $N_{f}^{i^{*}}$ under the dominant fatigue cycle i* is determined using the Morrow-corrected strain–life model. The ANN parameters are optimized by minimizing the mean squared error between the reference and predicted fatigue lives,

(15) $$L(\theta )=\frac{1}{n}\sum_{k=1}^{n}{\left(N_{f,k}^{i^{*}}-{\hat{N}}_{f,k}^{i^{*}}\right)}^{2}$$

where *n* is the number of training samples, and $N_{f,k}^{i^{*}}$ and ${\hat{N}}_{f,k}^{i^{*}}$are the reference and predicted fatigue lives of the *k*-th sample, respectively.

### 2.6. Reliability Assessment

After the surrogate model is constructed, it is coupled with Monte Carlo simulation to evaluate the fatigue reliability of the integrated blisk–shaft rotor under the dominant fatigue cycle. Based on the surrogate-predicted fatigue life, the fatigue damage of the dominant cycle is calculated as

(16) $$D_{i^{*}}\left(X\right)=\frac{n_{i^{*}}}{N_{f}^{i^{*}}(X)}$$

where $D_{i^{*}}\left(X\right)$ is the fatigue damage under the dominant cycle, $n_{i^{*}}$ is the applied cycle number of the dominant cycle, and $N_{f}^{i^{*}}(X)$ is the fatigue life predicted by the surrogate model.

(17) $$g(X)=N_{f}-N_{d}$$

where *N_f_* represents the fatigue life of the integrated blisk–shaft rotor under uncertain parameters, and *N_d_* denotes the required design life. Therefore, the failure probability can be expressed as

(18) $$P_{f}=P[g(X)\leq 0],$$

Using Monte Carlo simulation, the failure probability is estimated by

(19) $${\hat{P}}_{f}=\frac{1}{N}\sum_{j=1}^{N}I\left[g\left(X_{j}\right)\leq 0\right]$$

where *N* is the total number of Monte Carlo samples, $X_{j}$ is the *j*-th random sample, and *I*[⋅] is the indicator function defined as

(20) $$I\left[g\left(X_{j}\right)\leq 0\right]=\left\{\begin{array}{ll} 1, & g\left(X_{j}\right)\leq 0\\ 0, & g\left(X_{j}\right)>0 \end{array}\right.$$

The fatigue reliability is then obtained as

(21) $$R=1-{\hat{P}}_{f}$$

In addition to the failure probability and reliability, the surrogate-predicted fatigue life samples are used to characterize the probabilistic distribution of fatigue life under the dominant cycle. Statistical quantities are evaluated to quantify uncertainty-induced fatigue life dispersion. Furthermore, sensitivity analysis is performed based on the surrogate model to identify the dominant random variables affecting fatigue life and reliability.

## 3. Deterministic Fatigue Analysis and Critical Cycle Selection

### 3.1. Integrated Blisk–Shaft Rotor Model

**Figure 3:**
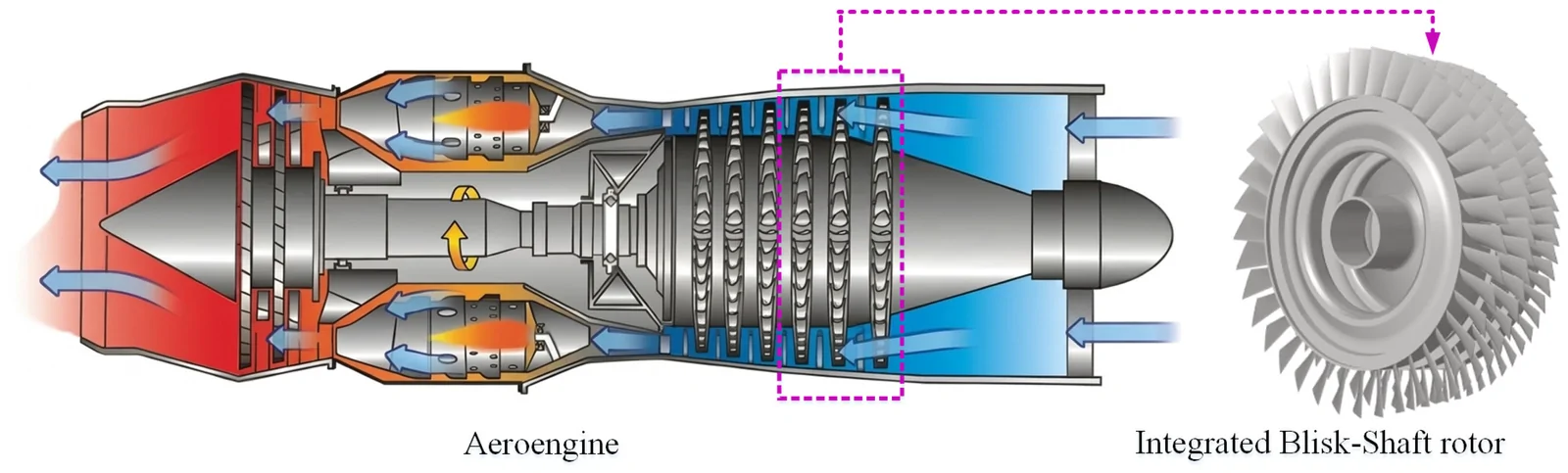
Structural configuration of the integrated blisk–shaft rotor in an aeroengine.

Figure 3 shows the structural configuration of the integrated blisk–shaft rotor investigated in this study. The rotor consists of three compressor blade–disk stages integrated with a central shaft; compared with conventional assembled blade–disk systems, this integrated configuration eliminates mechanical joints between the blades and disks, thereby improving structural compactness and load-transfer continuity. However, the strong structural coupling among the blade–disk stages and the shaft also increases the complexity of stress–strain analysis.

**Figure 4:**
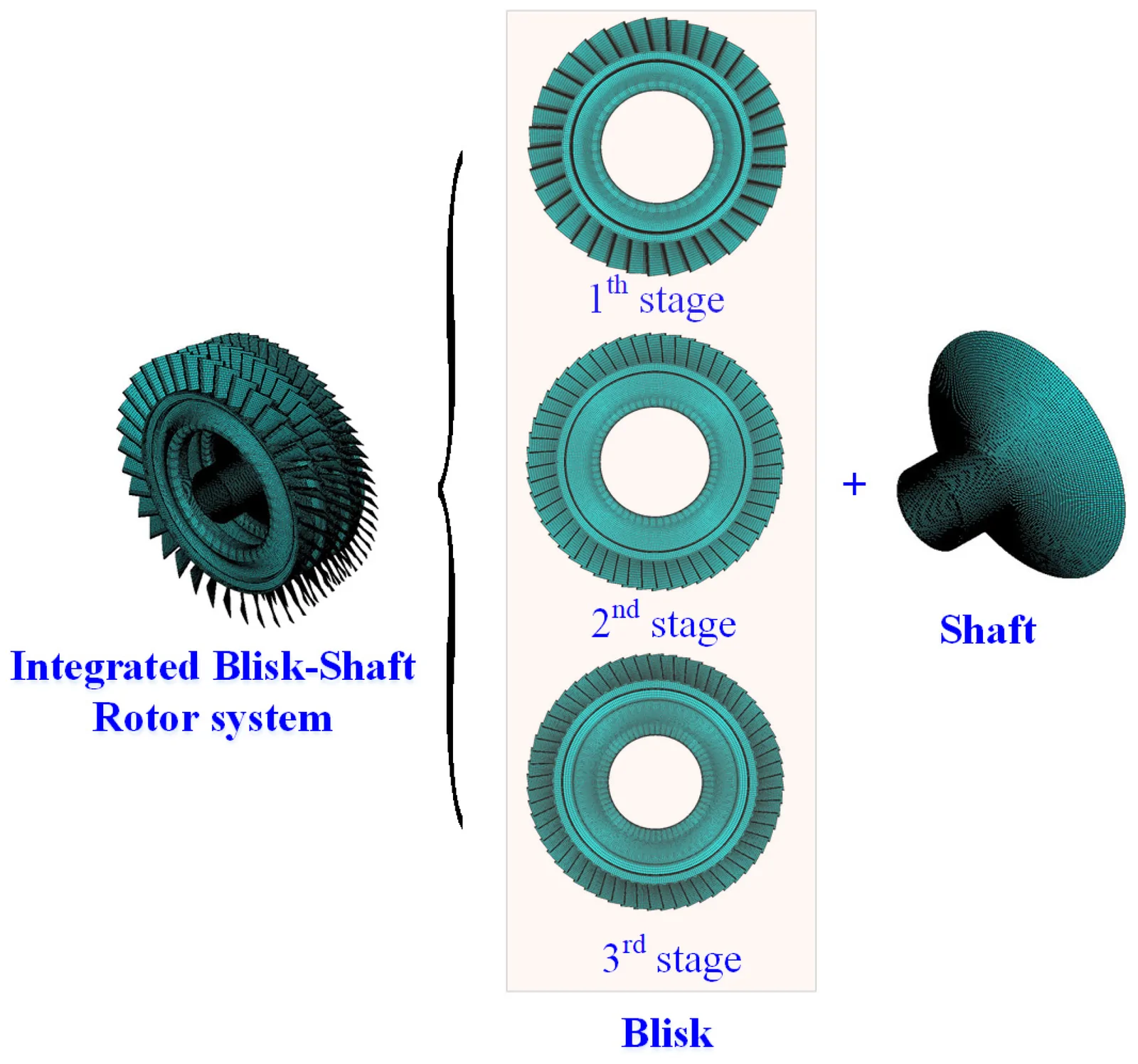
Substructure partition strategy of the integrated blisk–shaft rotor finite element model.

The three-dimensional finite element model of the integrated blisk–shaft rotor was established according to the geometric configuration shown in Figure 3. Owing to the differences in blade number and geometric characteristics among the three compressor stages, a substructure-informed modeling strategy was adopted, as illustrated in Figure 4. Each blade–disk stage was treated as an individual structural subdomain and subsequently assembled with the shaft substructure to construct the complete rotor model. At the interfaces between adjacent substructures, the corresponding interface nodes were geometrically coincident, and displacement compatibility was enforced to ensure continuous deformation and consistent load transfer across the substructure boundaries.

The rotor was discretized using SOLID185 and SOLID187 elements, with local mesh refinement applied to regions exhibiting pronounced geometric discontinuities and potential stress–strain concentrations, particularly the blade–disk transition regions, disk-root regions, and shaft-transition fillets. The resulting finite element model contained 576,917 elements and 826,686 nodes. This stage-wise discretization facilitates independent mesh control for blade–disk stages with different geometric features while enabling the accurate extraction of local stress–strain responses from candidate fatigue-critical regions.

The rotor material was assumed to be homogeneous and isotropic, and its mechanical properties are summarized in Table 1.

### 3.2. Boundary Conditions and Representative Load Cycles

The integrated blisk–shaft rotor is subjected to both centrifugal and aerodynamic loads during operation. In the finite element model, the boundary conditions were prescribed to represent the mechanical constraints of the rotor system. All degrees of freedom were constrained at the left-end bearing support, whereas the axial and radial degrees of freedom were constrained at the right-end drum, while the circumferential rotation is retained for applying the rotational speed.

Centrifugal loading is applied to the whole rotor body according to the prescribed rotational speed. Since centrifugal force increases with the square of rotational speed, it becomes the dominant source of stress at high-speed operating conditions. Aerodynamic loading is applied to the blade surfaces to represent the gas-flow-induced pressure acting on the blade–disk stages. For the *i*-th stage, the equivalent aerodynamic pressure can be approximated as

(22) $$P_{a,i}=P_{\mathrm{in},i}\frac{1+\alpha_{i}}{2}$$

where *P*_in,*i*_ is the inlet pressure of the *i*-th stage and α*_i_* is the pressure ratio between the inlet and outlet of the corresponding stage. The centrifugal body force acting on a material element can be expressed as

(23) $$dF=R\Omega^{2}\rho dV$$

where *R* is the radial distance of the material element, Ω is the angular velocity, *ρ* is the material density, and d*V* is the element volume.

**Figure 5:**
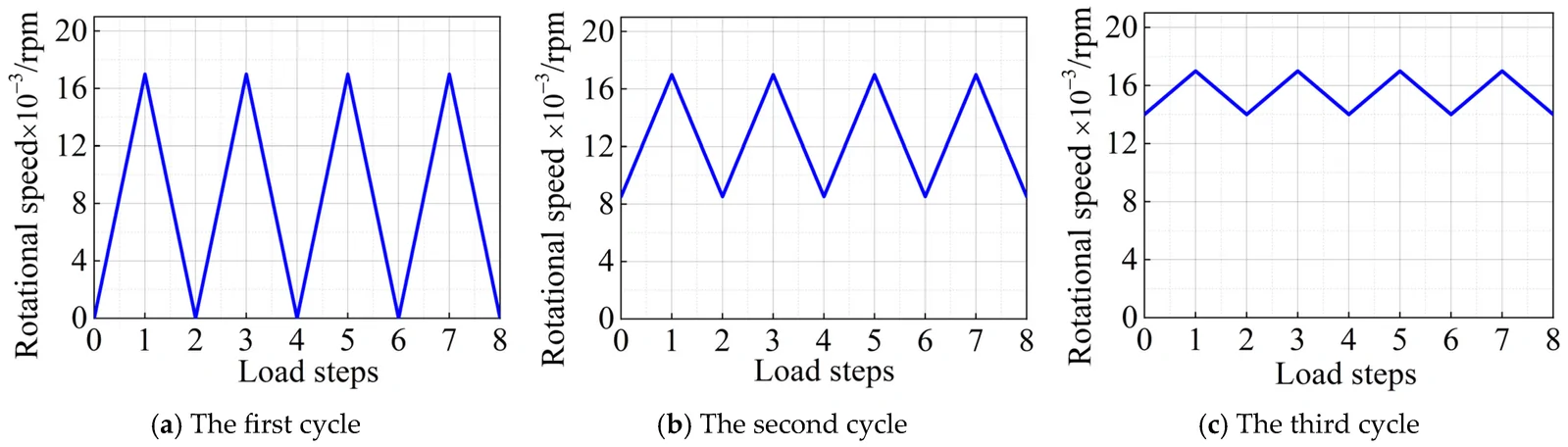
Representative operating load cycles of the integrated blisk–shaft rotor.

To evaluate the low-cycle fatigue behavior under representative operating transitions, three cyclic loading conditions are considered in the deterministic fatigue analysis. These cycles correspond to typical transitions among start-up, idle, cruise, and maximum continuous operating states. The representative operating load cycles are illustrated in Figure 5.

The first cycle, denoted as C1, corresponds to the start-up–maximum continuous–start-up transition. This cycle represents the largest speed variation and thus produces a high-amplitude fatigue loading condition. The second cycle, denoted as C2, corresponds to the idle–maximum continuous–idle transition, representing a medium-amplitude operating cycle. The third cycle, denoted as C3, corresponds to the cruise–maximum continuous–cruise transition, representing a typical service-related load fluctuation with a relatively smaller speed variation but a higher mean rotational speed.

The quantitative definitions of the three representative load cycles of the aeroengine over 1000 operating hours are listed in Table 2. These cycles are used for deterministic low-cycle fatigue analysis and damage contribution evaluation. The dominant fatigue cycle is then identified according to the damage contribution ratio and used for subsequent probabilistic reliability analysis.

### 3.3. Elastoplastic Stress–Strain Response and Critical Location

To identify the fatigue-critical region of the integrated blisk–shaft rotor, elastoplastic finite element analyses were first performed under the representative operating conditions defined in Section 3.2. Since centrifugal loading increases rapidly with rotational speed, the maximum continuous condition was selected as the primary operating condition for critical-location identification. Under this condition, the rotor experiences the highest centrifugal load and the most severe local stress–strain response.

**Figure 6:**
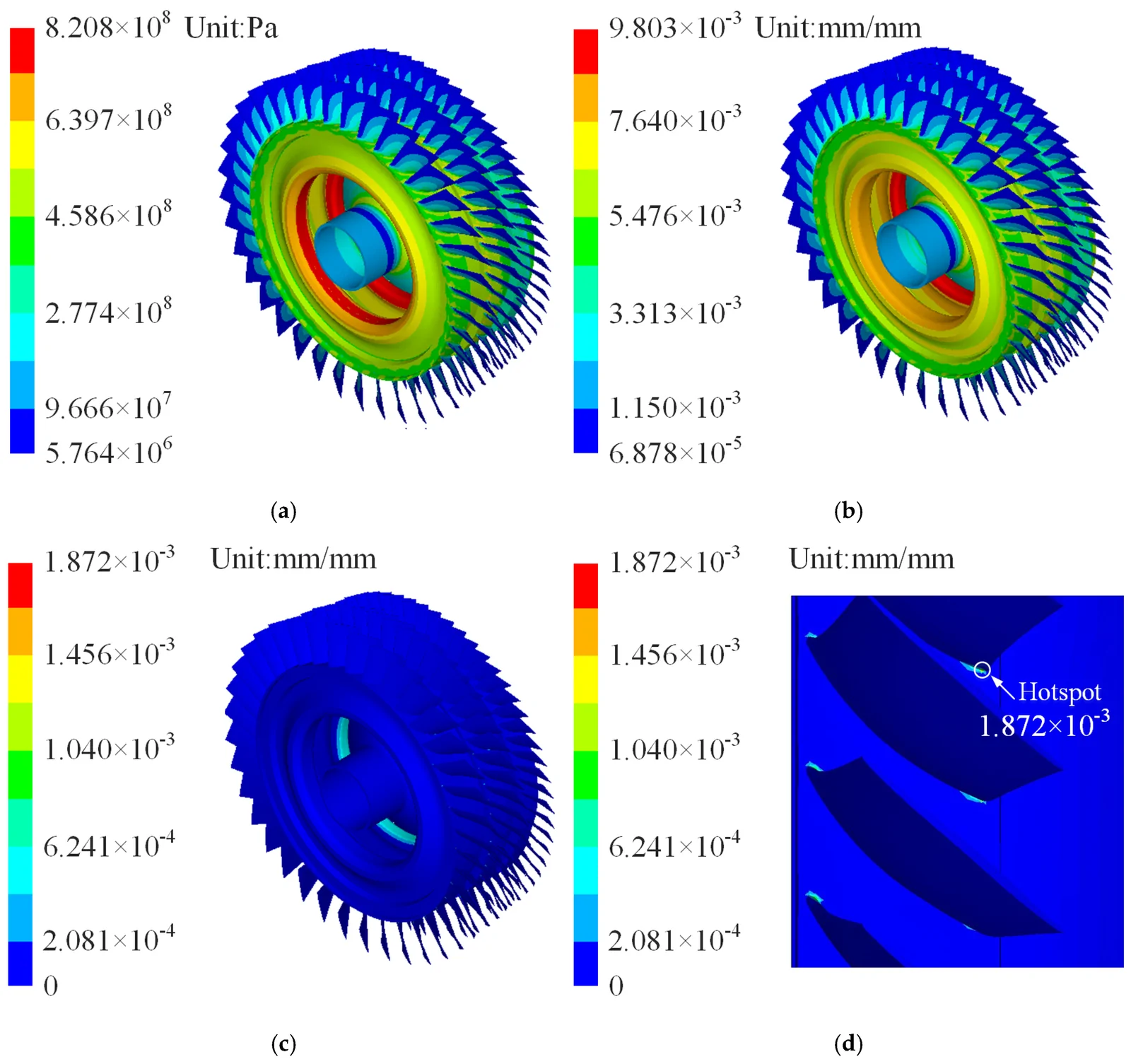
Elasto-plastic analysis results of blisk–shaft integration rotor in the maximum continuous state: (**a**) Von Mises stress distribution. (**b**) Equivalent strain distribution. (**c**) Equivalent plastic strain distribution. (**d**) Local equivalent plastic strain distribution and identified hotspot at the Stage 2 blade–disk transition region.

Figure 6a presents the von Mises stress distribution of the integrated blisk–shaft rotor under the maximum continuous operating condition. High-stress regions are mainly observed near the blade–disk transition and disk-root regions, with pronounced stress concentration at the blade–disk transition. This behavior is primarily attributed to the combined effects of centrifugal loading, geometric discontinuity, and local stiffness variation between the blade and disk. The corresponding equivalent strain distribution is shown in Figure 6b, exhibiting a concentration pattern generally consistent with the von Mises stress distribution. The maximum equivalent strain reaches 9.803 × 10^−3^ at the Stage 2 blade–disk transition region, indicating a more pronounced local deformation response than those in the other blade–disk stages and the shaft.

Relatively high stress and strain responses are also observed near the disk-root and shaft-transition regions owing to centrifugal loading and local geometric discontinuities. To quantitatively compare the responses of different rotor components, the maximum von Mises stress and equivalent strain of each blade–disk stage and the shaft are extracted and summarized in Table 3. The Stage 1 and Stage 3 blade–disk structures exhibit maximum von Mises stresses of 738.03 and 608.76 MPa, respectively, whereas the shaft shows a relatively lower stress response. In contrast, the Stage 2 blade–disk structure exhibits the most pronounced response, with a maximum von Mises stress of 820.87 MPa and a maximum equivalent strain of 0.00980.

To further characterize the localized plastic deformation at the identified critical region, the overall equivalent plastic strain distribution and the local hotspot at the Stage 2 blade–disk transition region are presented in Figure 6c,d, respectively. The equivalent plastic strain at the identified hotspot reaches 1.872 × 10^−3^, confirming pronounced localized plastic deformation. Since low-cycle fatigue behavior is strongly governed by the local strain response and associated plastic deformation, the Stage 2 blade–disk transition region is identified as the critical location for subsequent fatigue assessment.

To verify the mesh independence of the fatigue-critical response, a mesh-convergence study was conducted at the identified Stage 2 blade–disk transition hotspot using three progressively refined meshes containing approximately 0.328, 0.451, and 0.577 million elements, respectively. The equivalent strains at the hotspot were monitored as convergence indicators. With progressive mesh refinement, all three responses gradually approached stable values. For the three meshes, the variations in equivalent strain are only 0.57% and 0.02%. The finest mesh yielded a hotspot von Mises stress of 820.87 MPa and an equivalent strain of 0.00980. These results demonstrate the satisfactory mesh convergence of both the local elastoplastic response and fatigue life prediction. Therefore, the mesh containing 576,917 elements and 826,686 nodes was adopted for the subsequent fatigue and reliability analyses.

### 3.4. Low-Cycle Fatigue Assessment and Critical Cycle Selection

Based on the fatigue-critical location identified in Section 3.3, low-cycle fatigue (LCF) assessment is conducted at the blade–disk transition region of the second-stage blisk under three representative operating cycles. The fatigue life of the integrated blisk–shaft rotor is predicted using the Morrow mean-stress-corrected strain–life model described in Section 2. The nominal material fatigue parameters employed in the deterministic analysis are summarized in Table 4. For each operating cycle, the local stress amplitude, strain amplitude, and mean stress are extracted from the elastoplastic finite element results and subsequently incorporated into the strain–life model to determine the corresponding fatigue life.

The predicted fatigue responses under the three representative operating cycles are summarized in Table 5. Here, $\sigma_{em}$ denotes the equivalent mean stress of each operating cycle. It can be observed that significant differences exist among the fatigue responses corresponding to different operating transitions. For the start-up–maximum continuous–start-up cycle (Cycle 1), the predicted fatigue life is only 1.4510 × 10^4^ cycles, indicating the most severe cyclic deformation condition.

For the idle–maximum continuous–idle cycle (Cycle 2), although the maximum stress remains at the same level, the strain amplitude decreases to 0.003455 due to the reduced cyclic deformation range. As a result, the predicted fatigue life increases to 5.00308 × 10^5^ cycles. Compared with Cycle 1, the reduced strain amplitude significantly alleviates the accumulation of cyclic plastic deformation. For the cruise–maximum continuous–cruise cycle (Cycle 3), the strain amplitude decreases further to 0.00125, resulting in a substantially increased fatigue life of 6.60175 × 10^10^ cycles.

Although Cycle 1 exhibits the shortest fatigue life, the dominant fatigue cycle cannot be determined solely according to the fatigue life because the contribution of each cycle to the overall fatigue damage is also affected by its occurrence frequency during service operation. Therefore, the cumulative fatigue damage under the 1000 h operating spectrum is further evaluated using the Miner linear damage accumulation criterion.

According to the operating spectrum of the integrated blisk–shaft rotor, the total fatigue damage is calculated as

(24) $$D=\sum_{i=1}^{3}\frac{n_{i}}{N_{i}}=0.08683+0.00404+3.8399\times 10^{-7}\approx 0.09088$$

It can be observed that Cycle 1 contributes the majority of the total fatigue damage, while Cycle 2 and Cycle 3 contribute relatively small portions. Although Cycle 3 occurs most frequently during operation, its extremely long fatigue life results in a negligible damage contribution. Therefore, Cycle 1 is identified as the dominant fatigue cycle governing the fatigue degradation of the integrated rotor system. The identified dominant cycle is subsequently adopted as the critical operating condition for probabilistic fatigue reliability analysis in Section 4.

## 4. Probabilistic Fatigue Reliability Analysis

### 4.1. Random Variables for Probabilistic Analysis

In practical manufacturing and service conditions, the fatigue response of the integrated blisk–shaft rotor is affected by uncertainties in material properties, operating loads, and fatigue parameters. These uncertainties may lead to dispersion in local stress–strain responses and fatigue life, even when the same nominal operating cycle is considered. Therefore, selected parameters are treated as random variables in the probabilistic fatigue reliability analysis.

In this study, the random variables are divided into three categories: material parameters, loading parameters, and fatigue properties. Each random variable is assumed to be statistically independent and normally distributed [14,26]. For the *j*-th random variable, its probabilistic model is expressed as

(25) $$X_{j}\sim N\left(\mu_{j},\sigma_{j}^{2}\right)$$

where *μ*_j_ and *σ*_j_ are the mean value and standard deviation of *X_j_*, respectively. The coefficient of variation is defined as

(26) $$COV_{j}=\frac{\sigma_{j}}{\mu_{j}}$$

The random vector is written as

(27) $$X=\left[P_{1},\Omega ,E,\rho ,\sigma_{y},E_{t},\sigma_{f}^{\prime },b,\varepsilon_{f}^{\prime },c\right]$$

where $P_{1}$ is equivalent aerodynamic pressure, *E* is the elastic modulus, *ρ* is the material density, Ω is the rotational speed, $\sigma_{y}$ is the yield strength, $E_{t}$ is the tangent modulus, $\sigma_{f}^{\prime }$ and b are the fatigue strength coefficient and exponent, and $\varepsilon_{f}^{\prime }$ and c are the fatigue ductility coefficient and exponent, respectively. The statistical characteristics of the random variables of the integrated blisk–shaft rotor are listed in Table 6.

### 4.2. Probabilistic Fatigue Life Analysis

To quantify the influence of these uncertainties on fatigue performance, probabilistic fatigue life analysis is performed for the dominant fatigue cycle identified in Section 3.4. Since the start-up–maximum continuous–start-up cycle contributes the largest proportion of cumulative fatigue damage, only this representative operating cycle is considered in the subsequent probabilistic analysis, while the proposed framework can be readily extended to other operating conditions.

As shown in Table 6, Latin hypercube sampling (LHS) is employed to generate 1000 sets of random samples based on their prescribed probability distributions. For each sample, the local elastoplastic response at the critical blade–disk interface is combined with the Morrow strain–life model established in Section 2 to predict the corresponding fatigue life. The resulting fatigue life samples are then employed to investigate the probabilistic characteristics of the integrated blisk–shaft rotor.

**Figure 7:**
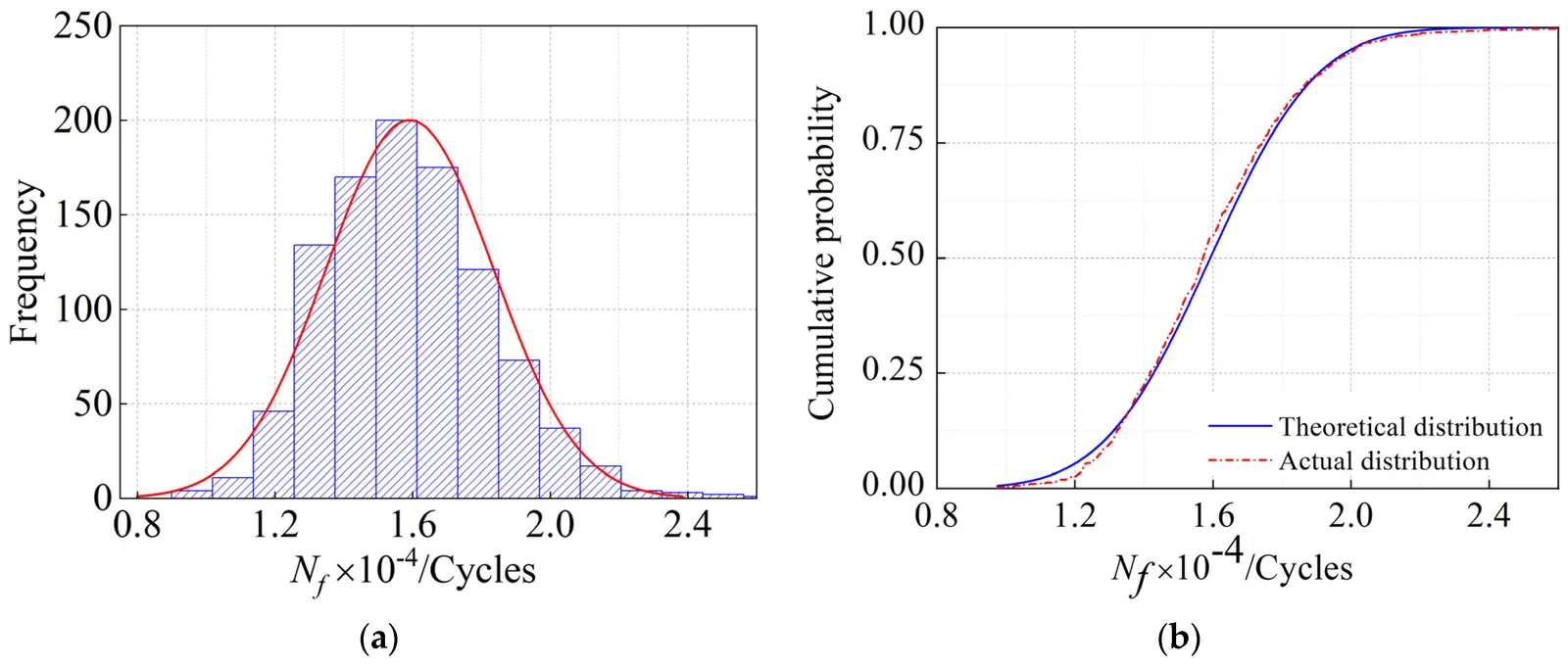
Probabilistic distribution of fatigue life for the integrated blisk–shaft rotor under the dominant fatigue cycle. (**a**) Probability density distribution (PDF). (**b**) Cumulative distribution function (CDF).

Figure 7a presents the histogram of the predicted fatigue life together with the fitted probability density function (PDF). It can be observed that the fatigue life exhibits an approximately normal distribution with noticeable statistical dispersion. The estimated mean fatigue life is 15,916 cycles, while the corresponding standard deviation is 2337 cycles, indicating that relatively small variations in material properties and operating parameters may result in significant fluctuations in fatigue life. This phenomenon further demonstrates the necessity of incorporating parameter uncertainties into fatigue reliability assessment.

Figure 7b presents a comparison between the empirical cumulative distribution function obtained from the fatigue life samples and the theoretical normal cumulative distribution function estimated using the sample mean and standard deviation. The close agreement between the two curves demonstrates that the predicted fatigue life exhibits an approximately normal distribution under normally distributed input variables.

To further examine whether parameter uncertainties alter the relative damage ranking among the representative operating cycles, the Miner damage associated with Cycles 1–3 was evaluated across the uncertainty samples. The statistical characteristics of the resulting fatigue damage are summarized in Table 7.

As shown in Table 7, the mean damage of Cycle 1 is approximately 22.4 times that of Cycle 2, whereas the contribution of Cycle 3 remains negligible. More importantly, the damage ranking *D*_1_ > *D*_2_ > *D*_3_ is preserved in 100% of the investigated uncertainty samples, demonstrating that the dominant role of Cycle 1 remains robust within the uncertainty ranges considered. Although Cycle 2 exhibits a finite secondary contribution, its mean damage is substantially lower than that of Cycle 1 and does not alter the cycle ranking across the uncertainty samples. Accordingly, Cycle 1 is adopted as the dominant-cycle approximation for the subsequent probabilistic reliability analysis. To enable efficient uncertainty propagation, an ICSO-optimized artificial neural network is employed as a surrogate for the computationally intensive finite-element fatigue analysis.

### 4.3. ICSO-ANN Surrogate Model Construction

As discussed in the previous section, probabilistic fatigue reliability analysis requires a large number of fatigue life evaluations under randomly generated input samples. The direct implementation of elastoplastic finite element analysis combined with strain–life calculation for each random sample would result in prohibitively high computational costs, making conventional Monte Carlo simulation inefficient for engineering applications. Therefore, an efficient surrogate model is developed to establish the nonlinear mapping between uncertain input parameters and fatigue life.

In this study, a feed-forward artificial neural network (ANN) is employed as the surrogate model because of its strong nonlinear approximation capability and computational efficiency. The uncertain material properties, operating parameters, and fatigue constants listed in Table 6 are selected as the input variables, while the predicted fatigue life under the dominant fatigue cycle is taken as the network output. Consequently, the ANN approximates the nonlinear relationship

(28) $$X=\left[P_{1},\Omega ,E,\rho ,\sigma_{y},E_{t},\sigma_{f}^{\prime },b,\varepsilon_{f}^{\prime },c\right]\to N_{f}$$

Although the conventional ANN has demonstrated excellent approximation capability, its prediction performance is highly dependent on the initial connection weights, thresholds, and network hyperparameters. Random initialization often leads to slow convergence and increases the possibility of premature convergence to local optima, which consequently degrades the prediction accuracy of fatigue life.

To overcome these limitations, an improved cat swarm optimization (ICSO) algorithm incorporating opposition-based learning (OBL) initialization is proposed to optimize the ANN hyperparameters.

#### 4.3.1. Basic Cat Swarm Optimization

Cat swarm optimization (CSO) is a population-based metaheuristic algorithm inspired by the behavioral characteristics of cats, and primarily comprises two search mechanisms: seeking mode and tracing mode. The seeking mode represents the local search behavior of cats, in which each individual explores its neighborhood by generating candidate positions around the current solution. The candidate solutions are evaluated using a predefined fitness function, and a promising candidate is selected to update the individual position. This mechanism primarily enhances the local exploitation capability of CSO and facilitates solution refinement.

In contrast, the tracing mode simulates the movement of cats toward promising regions guided by the current global best solution. Similar to the velocity-update mechanism in particle swarm optimization, the velocity and position of each individual are iteratively updated according to its current state and the global best position. This mechanism guides the population toward promising regions of the solution space and promotes convergence toward the optimum. The tracing process can be expressed as

(29) $$\left\{\begin{array}{l} v_{i}^{k+1}=v_{i}^{k}+cr\left(x_{\mathrm{best}\;}-x_{i}^{k}\right)\\ x_{i}^{k+1}=x_{i}^{k}+v_{i}^{k+1} \end{array}\right.$$

where $x_{i}^{k}$ and $v_{i}^{k}$ represent the position and velocity of the *i*-th individual at iteration *k*, respectively; $x_{\mathrm{best}}$ denotes the current global optimal position; *c* is the acceleration coefficient; and *r* is a random number within [0, 1].

Through the cooperation between seeking and tracing behaviors, CSO achieves a balance between exploration and exploitation during the optimization process.

#### 4.3.2. Opposition-Based Learning Initialization

Although CSO exhibits an effective optimization capability, the randomly generated initial population may not sufficiently cover the search space, resulting in slow convergence or premature convergence when solving complex nonlinear optimization problems. To overcome this limitation, an opposition-based learning strategy is introduced during population initialization. For a candidate solution *x_i_* within the search interval [*L*_i_, *U*_i_], its opposite solution is defined as

(30) $$x_{i}^{op}=L_{i}+U_{i}-x_{i}$$

where $x_{i}^{op}$ represents the opposite individual corresponding to $x_{i}$.

To provide a more comprehensive evaluation of the optimization performance of the proposed ICSO algorithm, four widely used benchmark functions with different characteristics, namely, Schwefel 2.21, Quartic, Sphere, and Schwefel 2.22, are selected for performance comparison. In addition to the original CSO, simulated annealing (SA) is introduced as an additional comparative algorithm. To reduce the influence of stochastic variability, each algorithm is independently executed 10 times for each benchmark function, and the mean and standard deviation of the final fitness values are used to evaluate the optimization accuracy and robustness. For CSO and ICSO, the population size and maximum number of iterations are set to 50 and 1000, respectively. The mathematical formulations and corresponding parameters of the benchmark functions are summarized in Table 8 [35].

**Figure 8:**
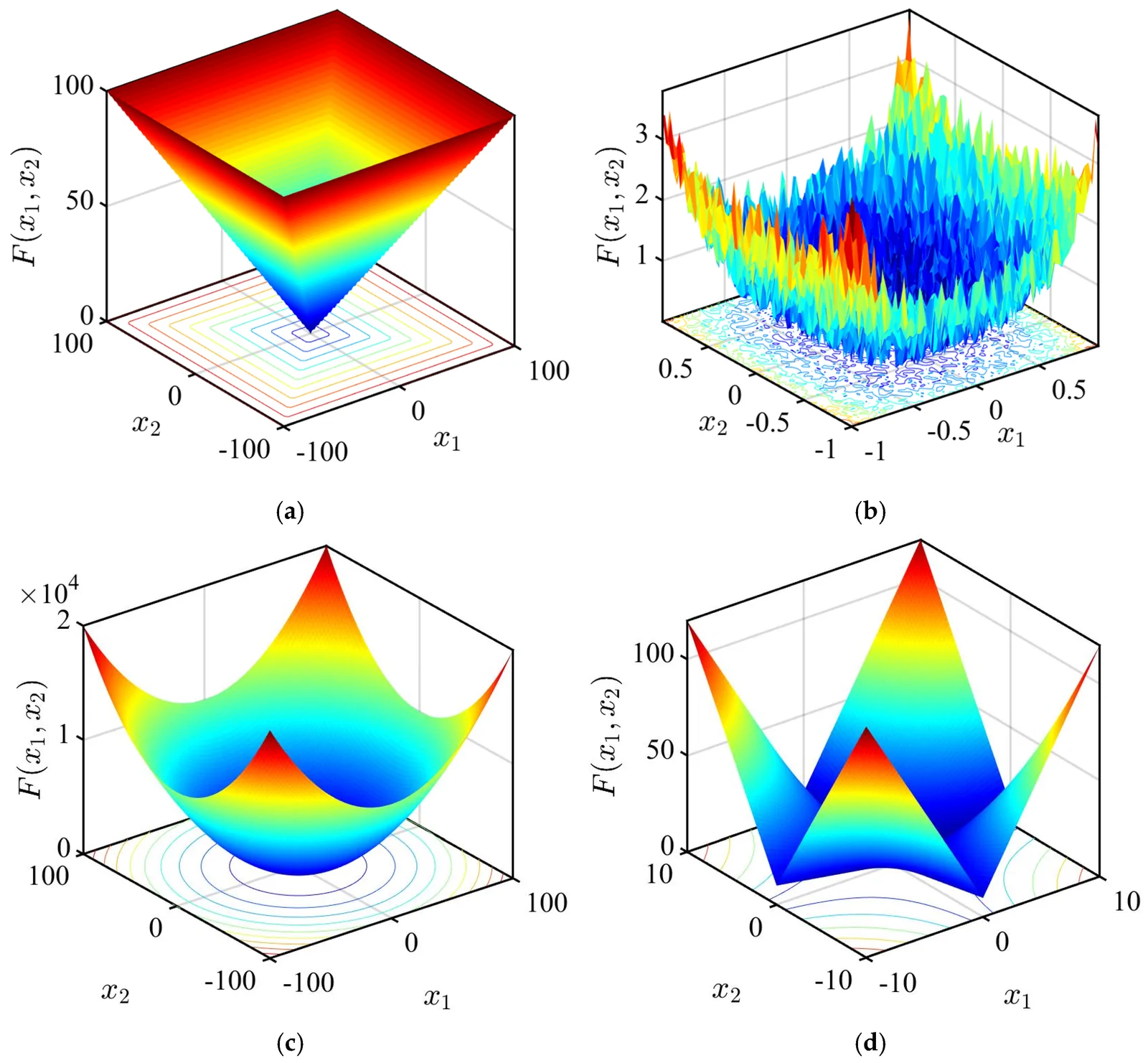
A 3D view of benchmark functions: (**a**) Schwefel2.21, (**b**) Quartic, (**c**) Sphere, (**d**) Schwefel 2.22.

**Figure 9:**
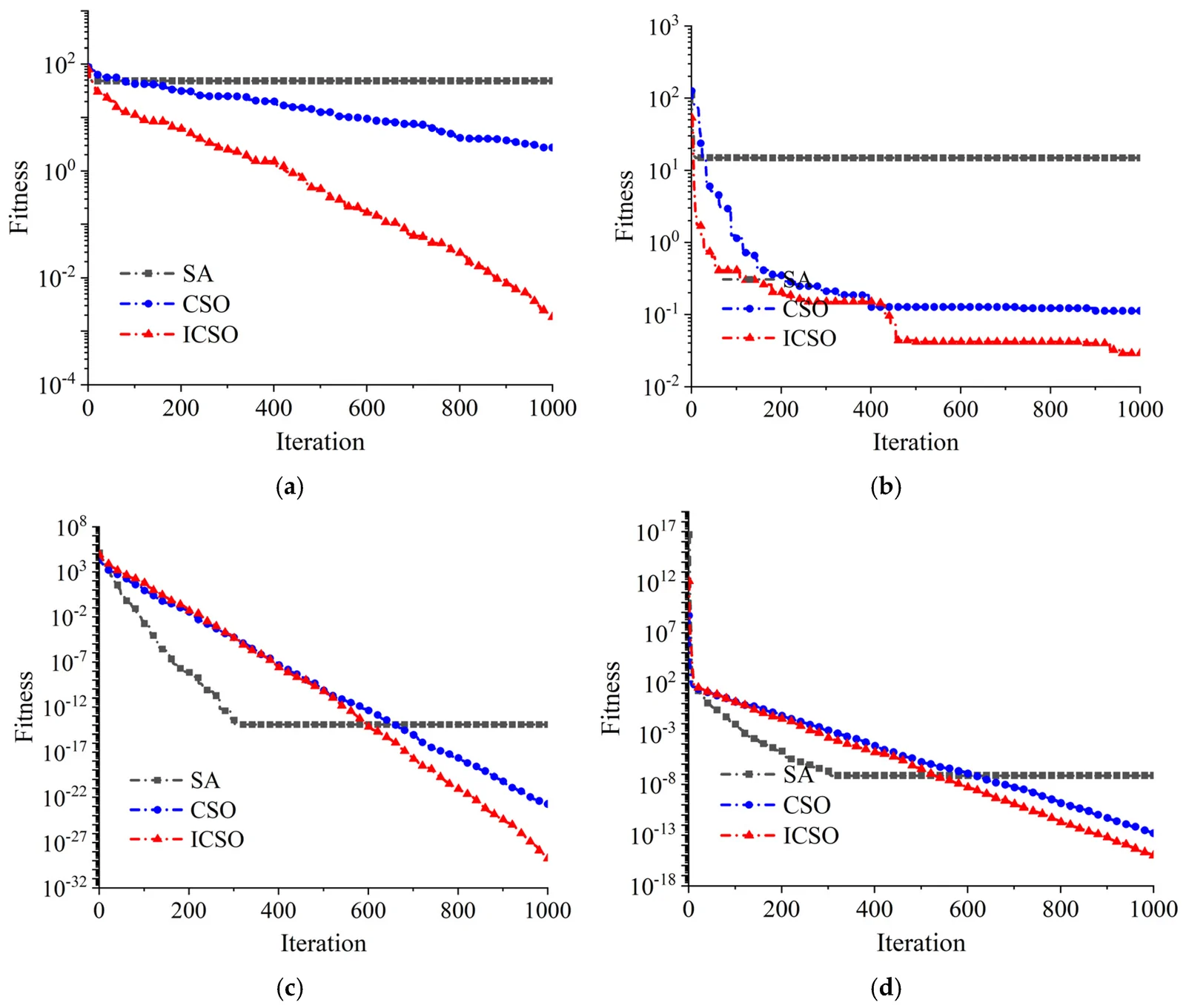
Convergence graphs of 3 algorithms: (**a**) Schwefel2.21, (**b**) Quartic, (**c**) Sphere, (**d**) Schwefel 2.22.

Figure 8 illustrates the three-dimensional landscapes of the four benchmark functions, while Figure 9 compares the convergence histories of SA, CSO, and ICSO for these functions. For the Schwefel 2.21 function, ICSO exhibits a sustained decrease in fitness throughout the iterative process, and reaches a substantially lower final fitness value than both CSO and SA, indicating improved convergence accuracy. For the Quartic function, ICSO also achieves a lower final fitness level, whereas SA rapidly stagnates and CSO shows limited improvement during the later iterations. For the Sphere and Schwefel 2.22 functions, ICSO ultimately attains the lowest fitness values, demonstrating its ability to maintain an effective search capability across benchmark functions with different characteristics.

To account for the stochastic nature of the optimization algorithms, Table 9 reports the mean and standard deviation of the final fitness values obtained from 10 independent runs. Across all four benchmark functions, ICSO consistently achieves the lowest mean fitness values, while its relatively small standard deviations demonstrate stable performance across repeated trials. Collectively, the convergence histories and statistical results confirm that, under the benchmark settings considered, ICSO offers superior convergence accuracy and robustness compared with conventional CSO and SA, thereby supporting its subsequent application to ANN parameter optimization.

#### 4.3.3. ICSO-Based ANN Parameter Optimization

Based on the superior optimization performance demonstrated above, the proposed ICSO algorithm is further integrated with ANN for fatigue life surrogate modeling. In the proposed framework, ICSO is employed to optimize the ANN model parameters for fatigue life prediction. The optimization variables are encoded as the decision vector of ICSO, including ANN connection weights and biases. The optimization objective is defined as minimizing the prediction error between the ANN output and the reference fatigue life obtained from the finite element-based fatigue analysis.

The ICSO -based hyperparameter optimization can be expressed as

(31) $$\theta^{*}=\arg\underset{\theta }{\min}L\left[f_{\mathrm{ANN}}(X;\theta ),N_{f}^{i^{*}}\right]$$

where **θ**^∗^ denotes the optimal parameter set obtained by ICSO. After optimization, the fatigue life surrogate model is written as

(32) $${\hat{N}}_{f}^{i^{*}}=f_{\mathrm{ICSO}-\mathrm{ANN}}\left(X\right).$$

### 4.4. Surrogate-Assisted Monte Carlo Reliability Analysis

Following the determination of the optimal ANN parameters using the proposed ICSO strategy, the resulting ICSO-ANN surrogate is validated against fatigue life data derived from finite element-based fatigue analyses. The surrogate employs a feedforward architecture consisting of 10 input neurons associated with the uncertain variables, a single hidden layer containing 15 neurons, and one linear output neuron representing fatigue life. Logistic sigmoid and linear activation functions are assigned to the hidden and output layers, respectively. Network training is performed using the BFGS quasi-Newton algorithm, with the mean squared error adopted as the loss function and without additional explicit regularization. Prior to training, both the input variables and fatigue life responses are scaled to [−1, 1] using min–max normalization.

**Figure 10:**
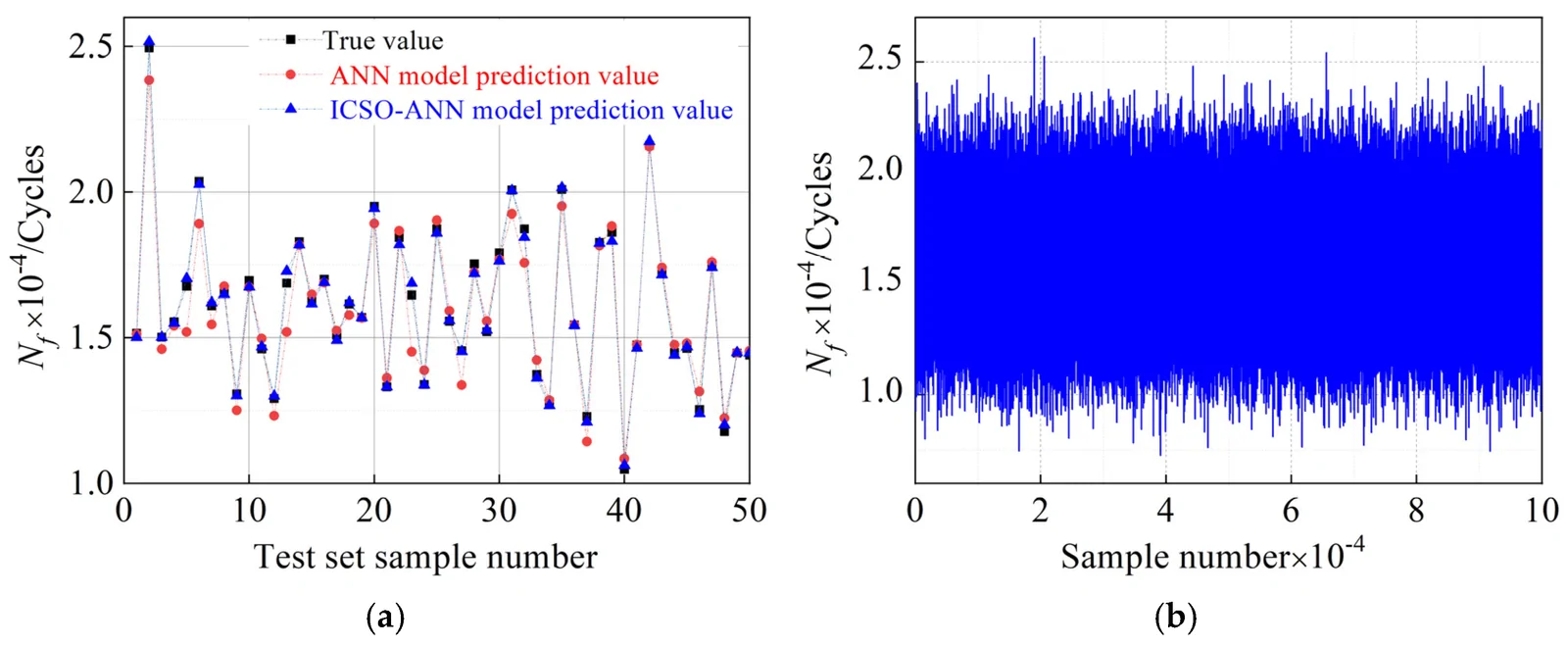
The ICSO-ANN surrogate model for fatigue life prediction of the integrated blisk–shaft rotor. (**a**) Comparison between predicted and finite element-based fatigue life results. (**b**) Fatigue life prediction under the dominant fatigue cycle.

A total of 1000 finite element samples generated through the fatigue analysis described in Section 4.2 are partitioned into four subsets of 250 samples, yielding four independent prediction assessments. For each assessment, 200 samples are used for model development, while the remaining 50 are reserved as a held-out test set for independent performance evaluation. To ensure a consistent and equitable comparison, the conventional ANN and ICSO-ANN are trained and evaluated using identical data partitions. The held-out test samples are strictly excluded from network training and ICSO optimization, and are used solely for final accuracy assessment. Figure 10a compares the finite element reference results with the predictions obtained from both surrogate models for one representative test set.

As illustrated in Figure 10a, the fatigue life predictions generated by both surrogate models generally reproduce the variation pattern of the finite element reference results across the held-out test set. Nevertheless, the ICSO-ANN predictions exhibit consistently closer agreement with the reference values than those obtained using the conventional ANN. Quantitatively, the MAPE decreases from 2.25% for the conventional ANN to 0.77% for the ICSO-ANN.

To further evaluate the overall predictive performance and reduce the influence of a single data partition, Table 10 summarizes the average error metrics obtained from the four independent prediction assessments. Compared with the conventional ANN, the ICSO-ANN reduces the average MAPE from 2.84% to 0.63%, the MAE from 457.20 to 125.01 cycles, and the RMSE from 648.10 to 161.97 cycles. Meanwhile, the coefficient of determination increases from 0.9428 to 0.9964, indicating substantially improved agreement between the surrogate predictions and the finite element reference results. Collectively, these results demonstrate that the ICSO-based optimization markedly enhances both the predictive accuracy and the generalization capability of the conventional ANN under the sampling and partitioning scheme adopted in this study.

Having established the predictive accuracy of the ICSO-ANN surrogate, the Monte Carlo sample size is increased to 10^5^ to characterize the probabilistic variation in fatigue life under parameter uncertainties. The random input realizations are generated using Latin hypercube sampling according to the prescribed probability distributions of the uncertain parameters. The validated ICSO-ANN surrogate is then used to predict the fatigue life of the integrated blisk–shaft rotor under the dominant fatigue cycle identified in Section 3.4, thereby avoiding repeated elastoplastic finite element analyses, as shown in Figure 10b, and the corresponding statistical parameters are summarized in Table 11.

The fatigue reliability is evaluated based on the fatigue life-based limit state function. Considering the inherent uncertainty associated with the design requirement of the integrated blisk–shaft rotor, the required design life *N_d_* is assumed to follow a normal distribution with a mean value of 11,000 cycles and a standard deviation of 121 cycles. Failure is assumed to occur when the actual fatigue life is lower than or equal to the required design life. Therefore, the limit state function can be defined as the difference between the predicted fatigue life and the design life, and the corresponding reliability is obtained as Equation (21). The fatigue life samples predicted by the ICSO-ANN surrogate model are subsequently combined with the design life samples to evaluate the reliability characteristics of the rotor system. The reliability results and computational efficiency comparison between the conventional Monte Carlo Method (MCM) and the proposed ICSO-ANN-assisted MCM are summarized in Table 12.

It can be observed that the reliability estimations obtained by the two methods show good agreement under different sampling sizes, indicating that the proposed surrogate-assisted approach maintains comparable accuracy to the direct MCM method. However, as the number of samples increases, the computational time required by direct MCM increases significantly due to the repeated execution of elastoplastic finite element fatigue analyses. In contrast, the ICSO-ANN-assisted MCM maintains a much lower computational cost while providing reliable fatigue probability estimations.

It should be noted that the reliability estimates obtained in this study are conditional on the assumed probability distributions and parameter ranges of the uncertain variables. Therefore, different uncertainty models or parameter ranges may lead to variations in the predicted reliability.

### 4.5. Sensitivity Analysis of Fatigue Life

#### 4.5.1. Parametric Influence Analysis Based on Response Surface

**Figure 11:**
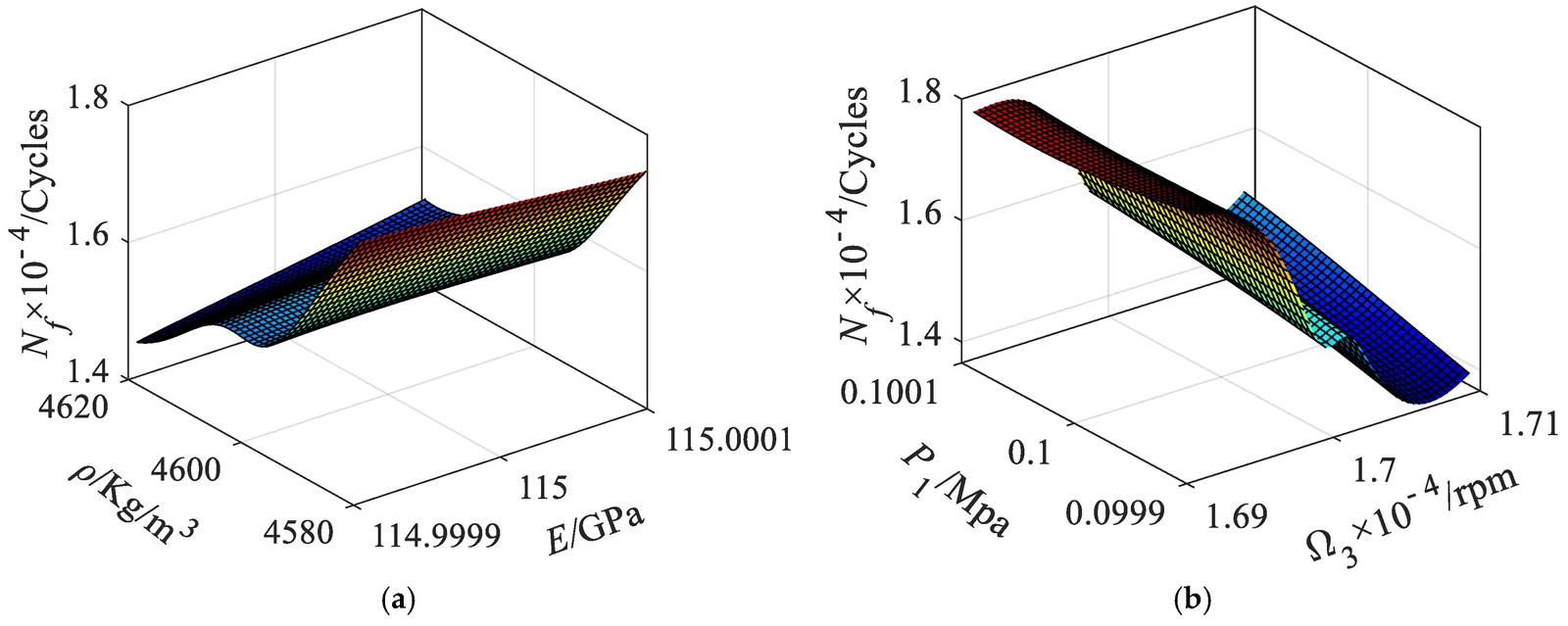

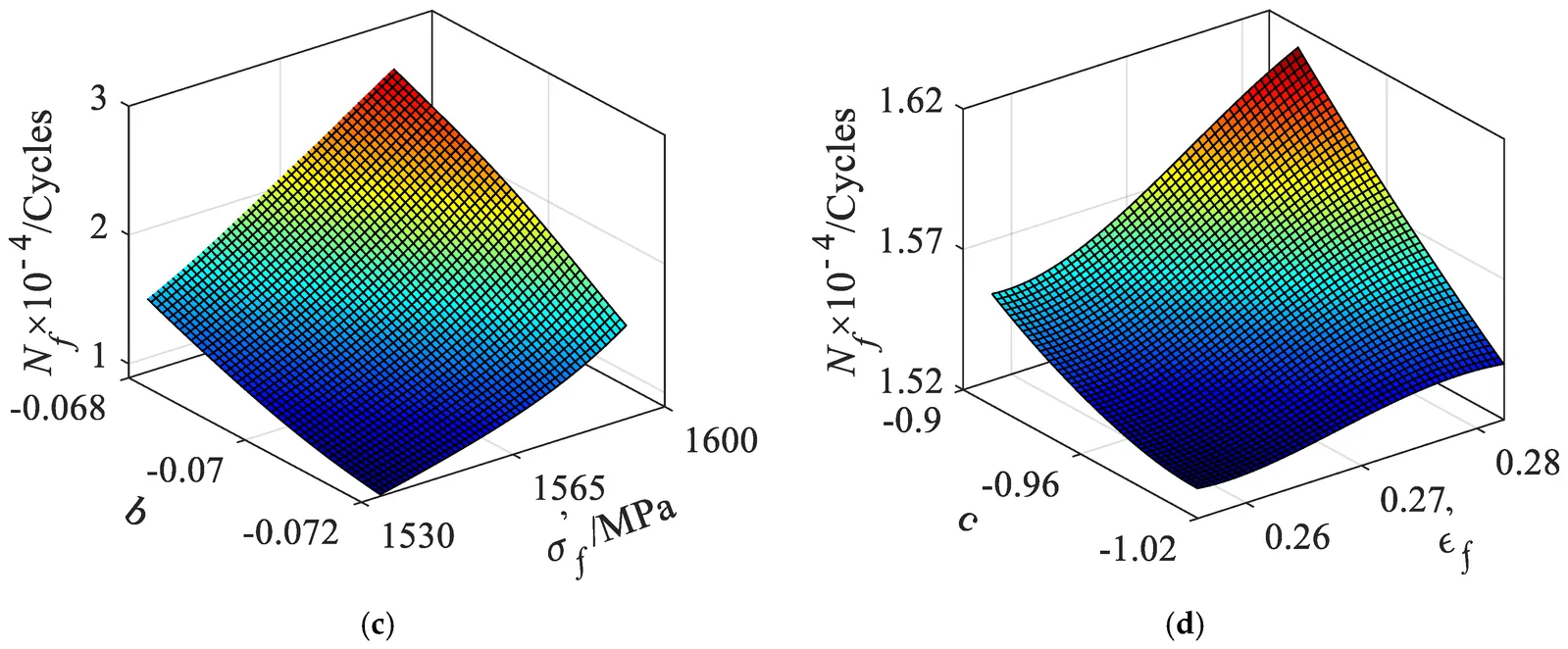
Effects of different parameters on fatigue life. (**a**) Elastic modulus and material density. (**b**) Aerodynamic pressure and rotational speed. (**c**) Fatigue strength parameters. (**d)** Fatigue ductility parameters.

To investigate the influence of uncertain parameters on the fatigue life of the integrated blisk–shaft rotor, the validated ICSO-ANN surrogate model is employed to perform response surface analysis. During the analysis, two input parameters are varied simultaneously within their prescribed ranges, while the remaining parameters are fixed at their nominal values. The fatigue life response surfaces obtained under different parameter combinations are presented in Figure 11. This approach enables the efficient evaluation of parameter effects without repeated nonlinear finite element fatigue analyses.

As shown in Figure 11a,b, material density and rotational speed exhibit a significant influence on fatigue life. Increasing density leads to a reduction in fatigue life due to the enhanced centrifugal loading during rotor operation, whereas the influence of elastic modulus is relatively limited. Similarly, fatigue life decreases markedly with increasing rotational speed, while aerodynamic pressure has a comparatively weaker effect. This indicates that centrifugal loading plays a dominant role in fatigue damage accumulation under high-speed operating conditions.

Figure 11c,d illustrate the effects of fatigue strength and fatigue ductility parameters on fatigue life. The fatigue life increases with an increasing fatigue strength coefficient and fatigue strength exponent, demonstrating their significant contribution to fatigue resistance. The fatigue ductility parameters also show a positive influence on fatigue life, but their effects are relatively weaker. Overall, the response surface analysis reveals that rotational speed and fatigue strength parameters are the dominant factors affecting fatigue life variation, providing a physical insight into uncertainty propagation and guidance for reliability-oriented design optimization.

#### 4.5.2. Global Sensitivity Analysis Based on Spearman Rank Correlation

Although the probabilistic analysis characterizes the dispersion of fatigue life under parameter uncertainties, it does not directly quantify the relative association between each uncertain input and the fatigue life response. Therefore, a sensitivity analysis based on Spearman rank correlation is conducted using the established ICSO-ANN surrogate model. Spearman rank correlation is a nonparametric measure that evaluates the strength and direction of the monotonic association between two variables based on their ranks. It does not require an explicit analytical response function or a specific distributional form, and is therefore suitable for the nonlinear input–output relationships considered in this study. The Spearman rank correlation coefficient between the *j*-th input variable and the fatigue life response is expressed as [36]

(33) $$R_{sj}=\frac{\sum_{i=1}^{m}\left({ℜ}_{ji}-{\bar{ℜ}}_{j}\right)\left({ℑ}_{i}-\bar{ℑ}\right)}{\sqrt{\sum_{i=1}^{m}{\left({ℜ}_{ji}-{\bar{ℜ}}_{j}\right)}^{2}}\sqrt{\sum_{i=1}^{m}{\left({ℑ}_{i}-\bar{ℑ}\right)}^{2}}}.$$

where *m* denotes the number of samples, and ${ℜ}_{ji}$ and ${ℑ}_{i}$ represent the ranks of the *j*-th input variable and the *i*-th observed value of the output variable, respectively. $R_{sj}$ represents the Spearman correlation coefficient between the *j*-th input variable and the structural response. A positive value of $R_{sj}$ indicates a positive correlation between the *j*-th input variable and the structural response, implying that the response increases with increasing input variable. Conversely, a negative value of $R_{sj}$ represents an inverse correlation, indicating that the structural response decreases as the corresponding input variable increases. The absolute value of $R_{sj}$ reflects the variation intensity of the structural response induced by changes in the *j*-th input variable, with a larger value indicating a stronger sensitivity.

To facilitate a quantitative comparison of the relative strengths of association among the input variables, the absolute Spearman rank correlation coefficients are normalized as

(34) $$P_{j}=\frac{\left|R_{sj}\right|}{\sum_{i=1}^{n}\left|R_{si}\right|}\times 100\%,j=1,2,\ldots ,n,$$

To further quantify the influence of uncertain parameters on the fatigue life variation of the integrated blisk–shaft rotor, the Spearman correlation coefficient (SCC)-based sensitivity analysis is performed using the established ICSO-ANN surrogate model. The fatigue life response is evaluated with respect to ten uncertain parameters, including rotational speeds $\Omega_{3}$, aerodynamic pressure $P_{1}$, elastic modulus E, material density ρ, yield strength $\sigma_{y}$, tangent modulus $E_{t}$, fatigue strength coefficient $\sigma_{f}^{\prime }$, fatigue strength exponent b, fatigue ductility coefficient $\varepsilon_{f}^{\prime }$ and fatigue ductility exponent c.

**Figure 12:**
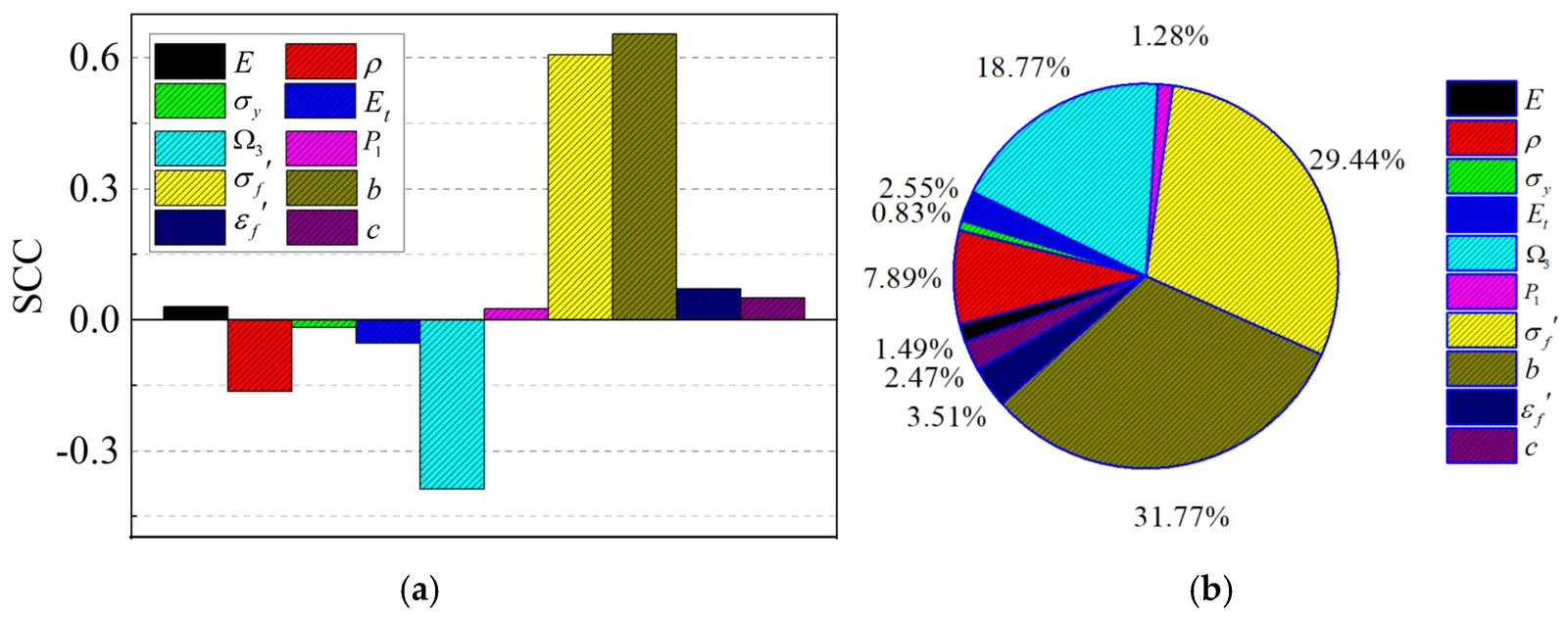
Fatigue life sensitivity distribution of the integrated blisk–shaft rotor. (**a**) Spearman correlation coefficients between uncertain parameters and fatigue life. (**b**) Relative sensitivity contributions of uncertain parameters to fatigue life.

As shown in Figure 12a, the uncertain parameters exhibit different sensitivity levels and correlation directions with respect to fatigue life. Among all parameters, the fatigue strength coefficient $\sigma_{f}^{\prime }$ and fatigue strength exponent *b* show strong positive correlations with fatigue life, indicating that an increase in material fatigue strength parameters can effectively improve the fatigue resistance of the rotor system. In contrast, rotational speed $\Omega_{3}$ and material density *ρ* exhibit negative correlations, suggesting that the increasing centrifugal loading caused by higher rotational speed and density accelerates fatigue damage accumulation and reduces fatigue life.

The normalized sensitivity contributions shown in Figure 12b further quantify the relative importance of each uncertain parameter. The fatigue strength exponent b and fatigue strength coefficient $\sigma_{f}^{\prime }$ contribute the largest proportions, accounting for 31.77% and 29.44%, respectively. The rotational speed $\Omega_{3}$ and material density ρ rank next, with contribution ratios of 18.77% and 7.89%, respectively. The remaining parameters make relatively limited contributions to fatigue life uncertainty. These results indicate that material fatigue parameters and rotational speed are the dominant sources of fatigue life uncertainty, and therefore should receive priority consideration in the reliability-oriented design and optimization of integrated blisk–shaft rotor systems.

## 5. Conclusions

An uncertainty-informed fatigue reliability assessment framework is developed for integrated blisk–shaft rotors by integrating elastoplastic finite element analysis, dominant fatigue-cycle identification, ICSO-ANN surrogate modeling, and Monte Carlo simulation. The elastoplastic analysis identifies the Stage 2 blade–disk transition region as the fatigue-critical location, where pronounced stress–strain concentration and localized plastic deformation occur under high-speed operating conditions. Combined with the Morrow strain–life relationship and Miner’s damage accumulation criterion, the deterministic multi-cycle analysis further identifies the dominant fatigue cycle, providing the basis for subsequent uncertainty propagation and reliability assessment.

An opposition-based learning strategy is incorporated into cat swarm optimization to improve the initialization and search performance of the ANN surrogate. Based on the finite-element dataset and independent test sets, the resulting ICSO-ANN achieves a mean absolute percentage error of 0.77%, demonstrating high accuracy in approximating the nonlinear relationship between uncertain parameters and fatigue life. The surrogate-assisted Monte Carlo analysis yields a fatigue reliability of approximately 98.6% for the prescribed design-life requirement. Moreover, on an identical 10^3^-sample computational basis, the proposed approach achieves an approximately 69-fold speedup over direct finite-element-based Monte Carlo analysis, substantially reducing the computational burden associated with repeated nonlinear simulations.

The uncertainty propagation and sensitivity analyses indicate that fatigue strength parameters and rotational speed are the dominant contributors to fatigue life variability within the investigated parameter ranges, whereas the remaining material and operating parameters exert comparatively limited influence. These results demonstrate that combining dominant-cycle reduction with surrogate-assisted probabilistic analysis provides an effective route for computationally intensive fatigue reliability problems in strongly coupled integrated rotor systems. The proposed framework offers a systematic basis for uncertainty quantification and the reliability-oriented assessment of integrated blisk–shaft rotors, and can support the structural integrity evaluation of advanced aeroengine rotor systems.

## Figures and Tables

**Table 1:** Material properties of the integrated disk–blade–shaft rotor.

Material Property	Value
Density (kg/m^3^)	4600
Elastic Modulus (GPa)	115
Poisson’s Ratio	0.3
Yield Strength (MPa)	820
Tangent modulus(GPa)	2.5

**Table 2:** A 1000 h load spectrum.

Cycle	Operating Transition	Number of Cycles in 1000 h	Speed Range/rpm
C1	Start-up–maximum continuous–start-up	1260	0–17,000–0
C2	Idle–maximum continuous–idle	2025	8500–17,000–8500
C3	Cruise–maximum continuous–cruise	25,350	14,000–17,000–14,000

**Table 3:** Elastoplastic response characteristics of different components of the integrated blisk–shaft rotor under the maximum continuous condition.

Response Parameter	Stage 1 Blade–Disk	Stage 2 Blade–Disk	Stage 3 Blade–Disk	Shaft
Maximum stress (MPa)	738.03	820.87	608.76	556.75
Location of maximum stress	Disk root	Blade–disk transition region	Disk root	Transition fillet region
Maximum equivalent strain	0.00841	0.00980	0.00720	0.007113
Location of maximum equivalent strain	Disk root	Blade–disk transition region	Disk root	Transition fillet region

**Table 4:** Fatigue parameters used for deterministic low-cycle fatigue analysis.

Parameter	$\sigma_{f}^{\prime }$	b	$\varepsilon_{f}^{\prime }$	c
Value	1564	−0.07	0.269	−0.96

**Table 5:** Stress–strain responses and predicted fatigue lives under different operating cycles.

	Cycle 1	Cycle 2	Cycle 3
$\sigma_{\max}$/MPa	820.87	820.87	820.87
$\sigma_{\min}$/MPa	0	217.18	580.95
$\varepsilon_{\max}$	0.00980	0.00980	0.00980
$\varepsilon_{\min}$	0	0.00289	0.00730
$\Delta \varepsilon_{\mathrm{eq}}/2$	0.00490	0.003455	0.00125
$\sigma_{em}$/MPa	410.435	519.025	700.91
$N_{i}$	14,510	500,308	66,017,496,759

**Table 6:** Statistical characteristics of each random parameter.

Variable	Distribution	Mean Value	Standard Deviation
Ω_3_/rpm	Normal	17,000	85
*P*_1_/MPa	Normal	0.1	0.0015
*E*/GPa	Normal	115	1.15
*ρ*/(k g/m^3^)	Normal	4600	46
*σ_y_*/MPa	Normal	820	12.3
*E_t_*/GPa	Normal	2.5	0.0125
$\sigma_{f}^{\prime \prime }$/MPa	Normal	1564	61.152
*b*	Normal	−0.07	4.9 × 10^−7^
$\varepsilon_{f}^{\prime }$	Normal	0.269	1.62 × 10^−5^
*c*	Normal	−0.96	2.07 × 10^−4^

**Table 7:** Statistical characteristics of fatigue damage for the three operating cycles under parameter uncertainties.

Statistical Measure	Cycle 1	Cycle 2	Cycle 3
Mean value	0.08099	0.00362	2.7551 × 10^−7^
Standard deviation	0.01214	0.00073	1.23885 × 10^−7^

**Table 8:** Test function.

Test Function	Name	Dim	Range	Optimum
$F_{1}(x)=\max_{1\leq i\leq D}\left|x_{i}\right|$	Schwefel2.21	30	[−100, 100]	0
$F_{2}(x)=\sum_{i=1}^{D}ix_{i}^{4}+\mathrm{random}[0,1)$	Quartic	30	[−1.28, 1.28]	0
$F_{3}(x)=\sum_{i=1}^{D}x_{i}^{2}$	Sphere	30	[−100, 100]	0
$F_{4}(x)=\sum_{i=1}^{D}\left|x_{i}\right|+\prod_{i-1}^{D}\left|x_{i}\right|$	Schwefel 2.22	30	[−10, 10]	0

**Table 9:** Comparison of the results of different algorithms for 4 benchmark functions.

		SA	CSO	ICSO
*F*_1_	Mean	58.69645	2.89126	0.000982
	Std	10.83242	0.139108	0.000604
*F*_2_	Mean	22.15345	0.14769	0.036753
	Std	10.36119	0.044117	0.005502
*F*_3_	Mean	7.63 × 10^−13^	8.89 × 10^−23^	8.37 × 10^−29^
	Std	1.05751 × 10^−12^	1.03278 × 10^−22^	6.11876 × 10^−29^
*F*_4_	Mean	8.09 × 10^−8^	7.35 × 10^−14^	7.44 × 10^−16^
	Std	1.34861 × 10^−8^	5.57672 × 10^−14^	2.33077 × 10^−16^

**Table 10:** Average predictive performance of the conventional ANN and ICSO-ANN over four independent assessments.

Surrogate Model	MAPE (%)	MAE (Cycles)	RMSE (Cycles)	*R* ^2^
Conventional ANN	2.84	457.20	648.10	0.9428
ICSO-ANN	0.63	125.01	161.97	0.9964

**Table 11:** Distribution parameter estimation results of fatigue life.

	Mean Value	Standard Deviation	Coefficient of Variation
Fatigue life/Cycles	15,924	2345.2	0.14727

**Table 12:** Comparison of reliability results and computational costs between direct MCM and ICSO-ANN-assisted MCM.

	Reliability		Time/s	
Sampling Number	MCM	ICSO-ANN-Assisted MCM	MCM	ICSO-ANN-Assisted MCM
10^2^	98%	99%	27,835	2944
10^3^	99.1%	98.9%	279,140	4048
10^4^	-	98.81%	-	5152
10^5^	-	98.6%	-	7360

## Data Availability

The original contributions presented in this study are included in the article. Further inquiries can be directed to the corresponding authors.
